# Time series DNA barcoding provides insight into factors influencing wood-boring and bark-feeding insect communities in Scots pine, Sitka spruce, and Noble fir stands

**DOI:** 10.1093/ee/nvad080

**Published:** 2023-08-17

**Authors:** Sophie de Becquevort, Niall J Mckeown, Max Blake, Paul W Shaw

**Affiliations:** Department of Life Sciences, Aberystwyth University, Aberystwyth, Ceredigion SY23 3DA, UK; Department of Life Sciences, Aberystwyth University, Aberystwyth, Ceredigion SY23 3DA, UK; Entomology, Forest Research, Alice Holt Lodge, Farnham, Surrey GU10 4LH, UK; Department of Life Sciences, Aberystwyth University, Aberystwyth, Ceredigion SY23 3DA, UK

**Keywords:** wood-boring, bark-feeding, DNA barcoding, larvae, conifer

## Abstract

Bark-feeding and wood-boring insect pests can have significant negative impacts on conifers and wood production. The damage they cause is expected to increase in the future due to climate change and the growth of international trade. This study employed DNA barcoding of beetle juveniles (Coleoptera) sampled from standing trap trees and cut log piles at regular intervals over a 2-yr period to monitor the beetle community dynamics and associated environmental factors. Tree species was found to have a major influence on beetle communities, most strikingly at the start of early decay stages. Lower species diversity was reported from standing trap tree samples compared to log pile samples, likely due to higher residual defences in dying and recently dead trees. While the species identified from standing trap trees are more likely to be a threat to the forestry sector, the species found in the log piles are more likely to be beneficial due to their high abundance and their ability to compete with pests for breeding substrate. The analysis of beetles collected inside trees revealed additional information on ontogenetic niches and host preferences beyond that acquired solely from flight interception trap data. Our results offer insights on community composition and dynamics of bark-feeding and wood-boring insect species in Welsh conifer forests and provide resources for monitoring and management of potential pest species.

## Introduction

Insects promote forest ecological succession by aiding in the elimination of dead, old, weak, stressed or diseased trees which benefits the overall health and resilience of the forest (Morris et al. 2018, Wermelinger 2021). Among insects, beetles (Coleoptera) are the most important decomposers in northern temperate regions. They can be phloem feeders (phloeophagous, bark-feeding beetles), wood feeders (xylophagous, wood-feeding beetles), or fungus feeders (fungiphagous, e.g., ambrosia beetles), but these categories sometimes overlap (Stokland et al. 2012). In the earliest stages of tree decomposition, insects feed on the phloem or the sapwood of live, weakened or recently dead trees (Wermelinger 2021). Early colonizing beetle species can be of particular importance in their impacts on forests, commercial forestry, and wood products. For example, bark beetles (Scolytinae) are capable of colonizing and killing living or weakened trees while feeding on the phloem (Sauvard 2004, Raffa et al. 2015). Beetle species that kill living trees or reduce the market value of stored timber are regarded as significant forestry pests and both native and invasive insect pest species can have a significant impact on forests.

The impact of pests on conifer forests can be economically significant because conifers are preferred over broadleaves in plantations and supply well over 50% of the world timber harvest (Farjon 2018). Moreover, the impact of pest species on natural and planted forests is likely to increase in the future as a consequence of climate change (Allen et al. 2010, Wainhouse and Inward 2016), which will have an impact on both the tree hosts and their associated insects. Firstly, more frequent and severe droughts will weaken conifers (Ray et al. 2010), leaving them more susceptible to pest attacks (Wainhouse and Inward 2016). Conifer monocultures, favored in forest management during the last decades, might be even more vulnerable to insect pest outbreaks (Williams et al. 2017, Huuskonen et al. 2021). Secondly, insect species are predicted to show changes in distributional range, seasonality and voltinism, and trophic interactions (Wainhouse and Inward 2016). Finally, pest outbreak frequency and intensity are also predicted to increase as outbreaks have been correlated with shifts in temperature and precipitation (Bentz et al. 2010).

There is a growing need to study tree and pest insect systems in order to predict the effects of climate change and the economic and environmental impacts of pests (Wermelinger 2021). In the United Kingdom, there is little information about the community ecology of bark-feeding and wood-boring pest species of conifers and their impact on forestry (Williams et al. 2017, 2021). Relationships between host tree species and their beetle community are usually inferred indirectly using flight interception traps. Indeed, while direct sampling of insects feeding on their breeding substrate provides precise knowledge on their nutrition requirements and ecological niches, it yields many larvae and pupae for which morphological-based identification to the species level is either challenging or impossible (Wu et al. 2017, O’Neill et al. 2018, Poland and Rassati 2019). As an alternative to dichotomous keys relying on distinct morphological features, DNA-based identification offers the possibility to identify specimens in various developmental stages without examination of morphological cues (Collins and Cruickshank 2013, Hendrich et al. 2015, Hoy 2018, Roe et al. 2019).

The present study aimed to depict beetle community structure, and its change in time over a 2-yr period, in Welsh coniferous forests through temporal sampling and DNA barcoding of larvae and pupae. A second objective was to investigate regional patterns and/or environmental variables driving community structure. The effects of tree species and early succession stages on beetle species composition (log pile vs. standing trap trees) were studied alongside the effects of log characteristics (log diameter, bark thickness, distance to the ground, and ground level) and sun exposure for the log pile. To our knowledge, this is the first study in Wales using larvae to determine links between beetle species, tree host, and environmental variables in fresh decaying wood communities of coniferous trees.

## Materials and Methods

### Tree Species and Site Selection

**Fig. 1.**
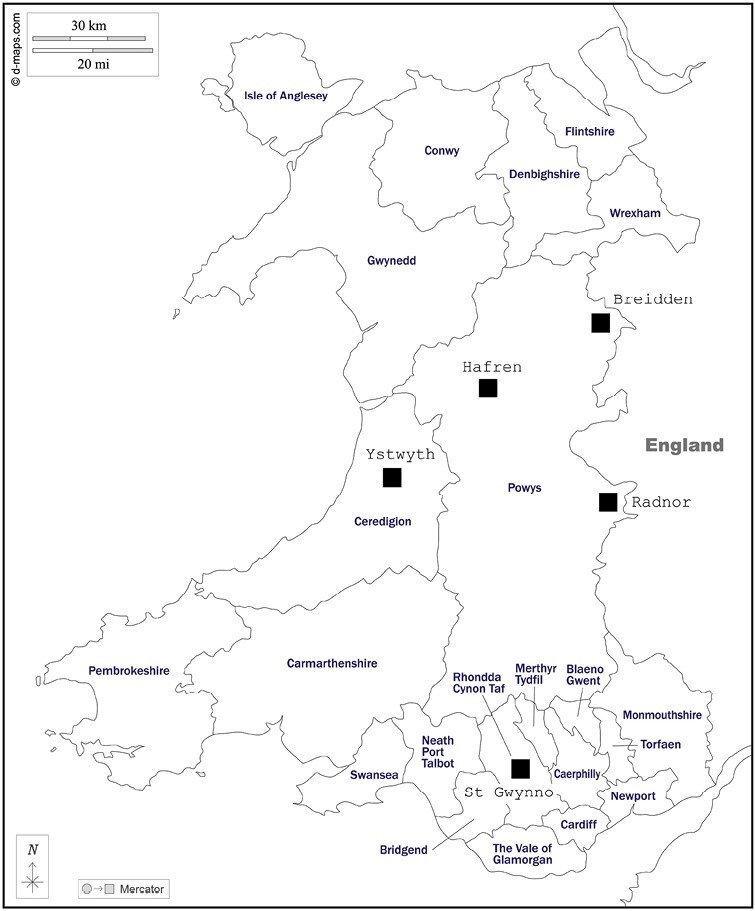
Locations of the 5 forestry sampling sites within Wales (Ystwyth, Hafren, Breidden, Radnor and St Gwynno).

**Fig. 2.**
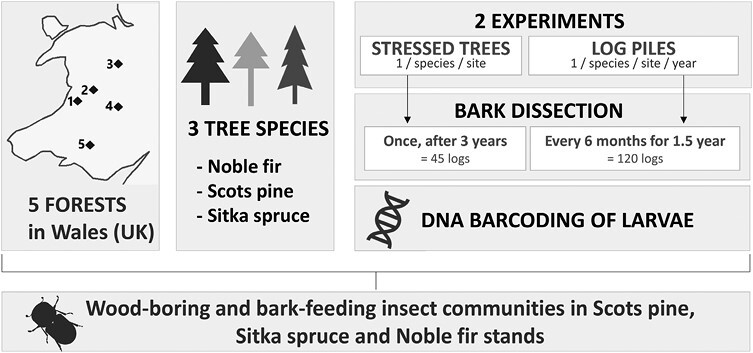
Experimental design of the study.

Three conifer species of different genera and commercially important in the United Kingdom were selected to provide a holistic view of insect community dynamics relevant to UK plantations: Sitka spruce (*Picea sitchensis*), Scots pine (*Pinus sylvestris*) and Noble fir (*Abies procera*). In November 2018, 5 Natural Resources Wales (NRW)-managed commercial forests were selected due to their geographical spread across Wales and the presence of the studied tree species with suitable planting dates. Within these forests and for each tree species, a monoculture stand was selected based on environmental similarities between stands (Fig. 1 and Table 1). Breidden forest was the only site where the 3 tree species were mixed into the same stand. The size of the 5 forest sites comprising the stands was on average of 1,846 ha, with a minimum of 253.2 ha (Breidden forest) and a maximum of 3,513 ha (Hafren forest). The size of each tree species stand ranged from 0.3 ha (St Gwynno forest, Scots pine stand) to 12.4 ha (Radnor forest, Sitka spruce stand), with an average of 5 ha. All stands were planted between 1940 (Ystwyth forest, Scots pine stand) and 1994 (Radnor forest, Noble fir stand), on average in 1962. Elevation ranged between 195 m (Ystwyth forest, Noble fir stand) to 485 m above sea level (Radnor forest, Noble fir stand), on average 328 m above sea level. This nested sampling design permitted disentangling of regional vs host tree effects and the sampling over successional stages for (i) standing trap trees (with some level of tree defenses) and (ii) log piles (without tree defenses) generated knowledge on host tree-pest insect systems (Fig. 2).

**Table 1.** Study site information for each of the three tree species

Forest	Forest size (ha)	Tree species	Site Code	Stand size (ha)	Year planted	Elevation (m)	Latitude	Longitude
Ystwyth	1,218	Sitka spruce	YST SS	10.8	1979	277	52.32515	−3.898272
		Scots pine	YST SP	8.3	1940	226	52.33074	−3.9205112
		Noble fir	YST NF	6.6	1954	195	52.34462	−3.8094049
Radnor	1,603	Sitka spruce	RAD SS	12.4	1982	446	52.28855	−3.1480417
		Scots pine	RAD SP	2	1951	379	52.2929	−3.129518
		Noble fir	RAD NF	3.7	1994	485	52.29012	−3.1987097
Hafren	3,513	Sitka spruce	HAF SS	2.3	1954	326	52.45414	−3.6279261
		Scots pine	HAF SP	1.1	1938	287	52.45598	−3.6335721
		Noble fir	HAF NF	1.2	1954	321	52.45227	−3.6253994
Breidden (Welshpool)	253	Sitka spruce	BRE SS	5	1960	304	52.71617	−3.0438112
		Scots pine	BRE SP	5	1960	307	52.71617	−3.0438112
		Noble fir	BRE NF	5	1960	309	52.71617	−3.0438112
St Gwynno (Llanwynno)	2,644	Sitka spruce	GWY SS	3.9	1965	422	51.67461	−3.4200601
		Scots pine	GWY SP	0.3	1951	299	51.68552	−3.4178988
		Noble fir	GWY NF	0.5	1982	332	51.65685	−3.4122328

### Insect Sampling

To capture the earliest stages of the insect succession, artificially stressed trees with lowered defenses were used to attract the few species that can overcome some level of tree defenses (Fettig and Hilszczański 2015, Krokene 2015, Holuša et al. 2017). One healthy tree per species and per site was selected and ring-barked in January 2019 by removing a 30 cm wide panel of bark from the lower part of the trunk with a chainsaw. An additional 30 cm of bark was removed in January 2020 as the trees were showing few signs of stress after 12 months (canopy intact, some trees showing strong growth from 2019). Trees were felled and sampled when they showed signs of insect activity (i.e., entry and exit holes or degraded bark), or when probing with a small chisel at eye level and below revealed evidence of insect or fungal activity under the bark. Insect activity on the upper portion of the trees was assessed using binoculars. After 30 months (summer 2021), all remaining trees were felled and sampled, whether they showed signs of insect activity or not. The whole trees were measured and cut into 1-m-long logs (including the bole in the crown). Beetle sampling was done on 3 logs that were picked randomly from each felled tree (1 at the bottom, 1 in the middle, and 1 at the top). Height on the tree of each log was recorded. Crown branches were out of the scope of the present study. For each log, the bark was removed and immature beetles (larvae, pupae, and young adults) were collected with tweezers from breeding galleries. Beetles from the same breeding gallery were preserved in 70% ethanol in individual tubes. All galleries found under the bark were sampled. Log diameter at both ends, before and after removing the bark, was recorded to calculate an average of log size and bark thickness.

Stacked piles of freshly cut logs were used to reproduce forestry conditions (i.e., stacked logs) and to investigate early colonizers of dead wood insect communities, as bark-feeding and wood-boring insect communities differ between dying and dead trees (Bertheau et al. 2009, Stokland et al. 2012). Three billet piles per tree species per site were set up in January 2019. Individual trees were felled, cut into 1-m logs and piled up in sunny areas as some species are known to prefer understory in open sunny positions (Lindhe et al. 2005, Stokland et al. 2012). For each site, 1 log pile per species was randomly selected and sampled in July 2019 (6 months), January 2020 (12 months), and July 2020 (18 months, by which time the phloem was unsuitable for insect breeding purposes). Scots pine sites were not sampled in July 2020 as the log bark was too decayed. At each sampling point, 3 logs (1 each of small, medium, and large diameter, respectively under 12 cm, between 12 and 28 cm, and above 28 cm of diameter) were selected randomly and carefully taken from the log pile to avoid disturbing the bark. Immature beetles were sampled from all galleries found under the bark in the same manner as for the standing trap tree experiment. Sun exposure (percentage of visible sky versus canopy cover) was calculated from in situ photographs with the program ImageJ, following the protocol “Canopy Closure from Digital Photos Using ImageJ” (Ecological Forester 2011).

### Insect Identification

DNA barcoding was used to identify larvae or pupae. A few teneral beetles (young adults) were found in breeding galleries alongside larvae and were identified based on morphological characteristics using the key from Duff (2016). Up to 12 individuals (each from a different gallery—randomly selected if more than 12 galleries were found on 1 log) were identified per log. Total genomic DNA was extracted following a CTAB-chloroform/isoamyl alcohol method (Winnepenninckx et al. 1993). A 710 base pair fragment of the mitochondrial COI gene was amplified using the Folmer DNA barcoding primers LCO1490/HCO2198 (Vrijenhoek 1994). Polymerase Chain Reaction (PCR) was performed with 2 μl of genomic DNA (ca. 2–10 ng of DNA), 0.5 μmol of each primer, 5 μl of 2 × BioMix (Bioline, UK), and 2 μl of ddH_2_O. PCR cycling conditions were: 95^o^C for 5 min, followed by 35 cycles of 95°C for 30 s, 1 min at 48°C annealing temperature, and 1 min 30 s at 72 °C, followed by a final extension stage at 72°C for 5 min. PCR products were then processed by Sanger Sequencing with the forward primer only. Specimen species identification was determined using the NCBI online Basic Local Alignment Search Tool (BLAST). Identifications were determined based on set thresholds for *e*-value (<0.001), query coverage (>80%), and sequence similarity (>95%). When a specimen sequence returned the same score for 2 species, the sequence was included in a Maximum Likelihood phylogenetic tree (Bootstrap method with 1,000 replications), using the model with the lowest BIC (Bayesian Information Criterion) score, drawn using the software MEGA (Kumar et al. 2018) using sequences from individuals with confirmed species ID from a reference database, to determine which species the specimen clustered with.

### Data Analysis

Species accumulation curves were obtained using the R package BiodiversityR (Kindt and Coe 2005) and used to assess the comprehensiveness of sampling for the log pile and standing trap tree experiments. For each log pile sampling time and for the standing trap trees results, the proportion of species was calculated as the number of species found in a log divided by the total number of species found in either all the standing trap trees or log piles. To characterize variation in abundance among species, the Shannon–Wiener diversity index and Simpson’s index of diversity (1-Simpson’s Index) were calculated. Response-transformation extensions were performed on the different indices using the R package emmeans (Lenth (2019): Arcsin-square-root (parameter *p* equal to 100) transformation was used on species proportions and generalized logarithmic transformation (log(*y* + *p*); *p* = 1) was used on Shannon–Wiener and 1-Simpson’s indices. The result of these transformations was then used as an environment in which linear mixed models (estimated using REML and nloptwrap optimizer) were fitted using the lmer function in the lme4 package for R (Bates et al. 2015). The 2 experiments (i.e., standing trap tree and log pile) were compared using a model including “Tree Species” and “Experiment Type” as fixed factors and “Forest Site” as a random effect. To evaluate the effect of the type of experiment, a depleted model with the latter removed was compared to a model with both factors but any interaction removed (to avoid including the contribution of the interaction) using an Analysis of Variance (ANOVA). Analysis of the standing trap tree experiment results was done as a whole (i.e., as all samples were representing the same successional stage) using a model including “Tree Species” as fixed factor and “Forest Site” as a random effect. Finally, the log pile experiment results were analyzed in the same way, but 1 model was fitted for each sampling period (i.e., Summer 2019, Winter 2020, and Summer 2020). When a factor was significant, back-transformed estimated marginal means were computed with the R package emmeans (Lenth 2019).

The environmental variables structuring the communities were analyzed for the 2 experiments. The relationships between species composition and underlying environmental gradients were investigated based on the compositional resemblance or dissimilarity between log samples, using ordination and numerical classification analyses (Borcard et al. 2018). The function “Rankindex” from the R package vegan was used to compare the Bray–Curtis dissimilarity Index, most commonly used on abundance data, to 2 alternatives (Binomial and Cao indices) which were developed to compare species abundances with moderate to large differences (Borcard et al. 2011). The function metaMDS of the picante/vegan R package was used to perform NMDS and find a stable solution (convergence of stress) using several random starts for both 2 and 3 dimensions (Kembel et al. 2010, Oksanen et al. 2020). The iteration process stopped when convergence was found or when the maximum number of 250 random starts was reached. The maximum number of iterations in a single NMDS run was 999. Assessment of the stress scatter around the fitted line in the stress plots (plot of ordination distances against original dissimilarities) and overall stress values were used to select which dimension to keep for analysis.

The R functions ordiplot and ordihull were used to plot the ordination results (samples and insect species) and polygons connecting the outermost site scores of the different factors (tree species, forest site, and sampling time). The function envfit was used to fit environmental variables (Samples × Environmental variables) onto the ordination plot to represent how they were correlated with the ordination axes generated from the community data (species × samples; Oksanen et al. 2020). The environmental variables were presented as arrows (lengths proportional to the correlation between ordination and environmental variables; directed toward the steepest increase in the environmental variable value). To quantify the relationship between dissimilarity measures and different explanatory variables (categorical or continuous variables) using permutational multivariate analysis of variance (MANOVA), the function Adonis was used (999 permutations, Oksanen et al. 2020). A classification method was also used to separate samples into a limited number of groups (clusters) containing similar samples. Distances between the samples were calculated using binomial distribution and samples were clustered using an agglomerative hierarchical clustering algorithm with the hclust function in R (R Core Team 2021).

## Results

### Beetle Species Identified From the 2 Experiments

**Fig. 3.**
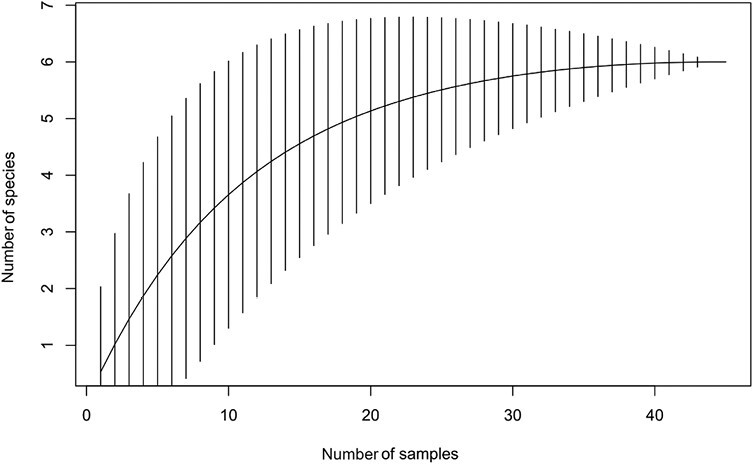
Species accumulation curve for the standing trap tree experiment.

**Fig. 4.**
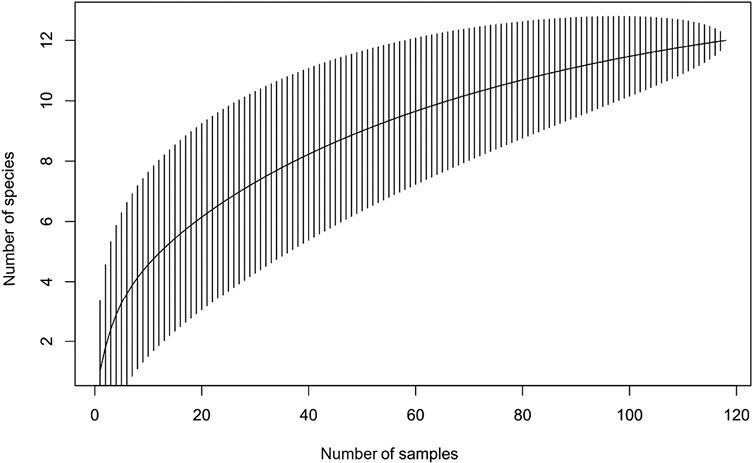
Species accumulation curve for the log pile experiment.

A total of 126 beetles were identified from 45 logs in the standing trap trees experiment and a total of 525 beetles were identified from 119 logs in the log piles experiment (combined across sites and tree species – 1 log pile did not have a suitable medium log). On average, the number of galleries found on each log was 6.025. It was 8.451 for the log piles and 3.600 for the standing trap trees. The species accumulation curve for the standing trap tree experiment (Fig. 3) showed flattening whereas for the log pile experiment the plateauing of the species accumulation curve was less obvious (Fig. 4).

**Fig. 5.**
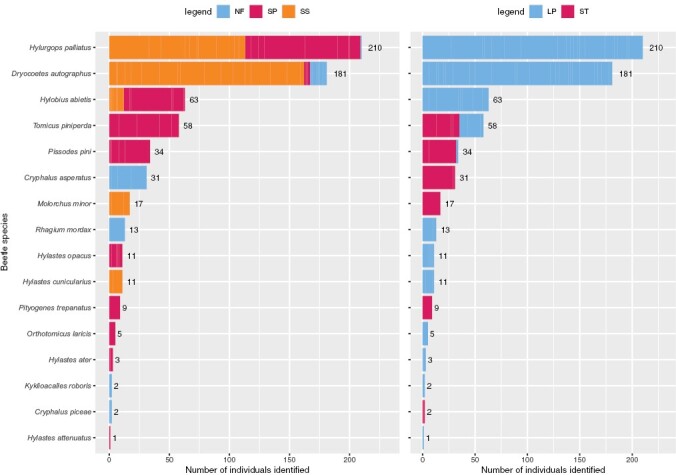
Abundance of 16 identified beetle species found in the 3 tree species (left) and in log piles or Standing trap trees (right). Tree Species: NF = Noble fir, SS = Sitka spruce, SP = Scots pine. Experiment: LP = Log piles, ST = Standing trap trees.

The number of beetles identified from log piles decreased over successive sampling: 254 in Summer 2019; 198 in Winter 2019; 73 in Summer 2020. From 651 individuals sampled, 16 beetle species were found (Fig. 5). *Hylurgops palliatus* (Gyllenhal) and *Dryocoetes autographus* (Ratzburg) were the 2 most abundant species (60% of the beetles identified, respectively 32 and 28%) and were the only species found in logs from all 3 tree species (in the log pile experiment only, Fig. 5). Two beetle species were found in logs from both the standing trap tree and log pile experiments (*Tomicus piniperda* (Linnaeus) and *Pissodes pini* (Linnaeus)) which accounted for 9 and 5% respectively of the beetles identified and were collected only from Scots pine logs (Fig. 5). Of the other beetle species all but *Hylobius abietis* (Linnaeus, found in Socts pine and Sitka spruce log piles) exhibited an association with a particular tree species and type of susbtrate: 5 species in Scots pine, 4 species in Noble fir and 2 species on Sitka spruce; 4 species were found exclusively in standing trap trees and 10 species only in log piles. Noble fir supported 10% of the number of individual beetles identified, Scots pine 42%, and Sitka spruce 48%. Geographically, 15% of beetle individuals were identified from St Gwynno, 20% from Breidden, 20% from Hafren, 21% from Radnor and 24% from Ystwyth. The most abundant species (*H. palliatus*, *D. autographus,* and *H. abietis*) were found on all 5 forest sites, and a similar pattern can be seen for *P. pini* and *Hylastes opacus* (Erichson) which were found on 4 out of 5 forest sites. The other species were found on half or less of the forest sites.

### Comparison of Log Piles and Standing Trap Trees

For Shannon–Wiener diversity index, 1-Simpson’s index of diversity and Species Proportion, the interaction between Tree Species and Experiment Type was significant (respectively χ²(2) = 7.463, *P* = 0.024; χ²(2) = 7.112, *P* = 0.029; χ²(2) = 17.242, *P* < 0.001). The type of experiment had significant effect on the 3 diversity measures, with Log piles showing higher values than standing trap trees (respectively: β = 0.100, SE = 0.038, χ²(1) = 6.732, *P* = 0.009; β = 0.065, SE = 0.026, χ²(1) = 6.066, *P* = 0.014; β = 0.004, SE = 0.004, χ²(1) = 9.410, *P* = 0.002). Similarly, Tree species also had a significant effect (respectively χ²(2) = 26.685, *P* < 0.001; χ²(2) = 26.257, *P* < 0.001; χ²(2) = 37.461, *P* < 0.001). All diversity measures were significantly higher in Sitka spruce (β = 0.089, SE = 0.045, *t*(164) = 4.719, *P* < 0.001; β = 0.063, SE = 0.031, *t*(164) = 4.623, *P* < 0.001; β = 0.008, SE = 0.003, *t*(164) = 6.762, *P* < 0.001) and Scots pine (β = 0.190, SE = 0.047, *t*(164) = 4.564, *P* < 0.001; β = 0.134, SE = 0.032, *t*(164) = 4.682, *P* < 0.001; β = 0.014, SE = 0.003, *t*(164) = 5.901, *P* < 0.001) than Noble fir. On the other hand, diversity measures were not significantly different in Scots pine and Sitka spruce (*P* = 0.95, *P* = 1, and *P* = 1).

### Beetle Communities in Standing Trap Trees

Tree species had a significant impact on the beetle diversity in standing trap trees. Shannon–Wiener diversity index, 1-Simpson’s index of diversity and Species Proportion were significantly lower in Sitka spruce than in Scots pine (respectively β = −0.164, SE = 0.054, *t*(45) = −−3.023, *P* = 0.009; β = −−0.123, SE = 0.040, *t*(45) = −3.087, *P* = 0.009; β = −0.022, SE = 0.008, *t*(45) = −2.749, *P* = 0.018). On the other hand, diversity measures were not significantly different between Noble fir and Sitka Spruce (respectively β = −0.034, SE = 0.054, *t*(45) = −0.620, *P* = 0.539; β = −0.019, SE = 0.040, *t*(45) = −0.476, *P* = 0.636; β = −0.008, SE = 0.008, *t*(45) = −0.963, *P* = 0.341). Finally, Shannon–Wiener diversity index and Simpson’s index of diversity were significantly lower in Noble fir than Scots pine standing trap trees (respectively β = −0.131, SE = 0.054, *t*(45) = −2.404, *P* = 0.042; β = −0.104, SE = 0.040, *t*(45) = −2.611, *P* = 0.025) but not Species proportion (β = −0.014, SE = 0.008, *t*(45) = −1.786, *P* = 0.164).

**Fig. 6.**
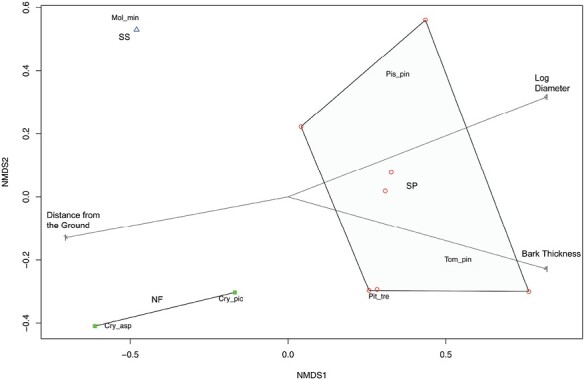
Nonmetric multidimensional scaling (NMDS) plot generated using species composition within log samples from standing trap trees and showing similarities/dissimilarities between log samples (Triangles = Sitka spruce logs, Squares = Noble fir logs, Circles = Scots pine logs). Environmental variables were added and shown with arrows. The stress value of the analysis is 0.07.

Differences in beetle species composition between standing trap tree samples are represented in Fig. 6, with samples from each tree species identified by polygons (maximum area of the score of each tree species group in the ordination). The plot showed no overlapping polygons. The ordination plot also illustrates the association between some beetle species and particular environmental factors (e.g., *Molorchus minor* (Linnaeus) and *Cryphalus asperatus* (Gyllenhal) with small log diameter, small bark thickness, and high distance from the ground). Supplementary Table 1 shows the range of values for the continuous variables.

**Fig. 7.**
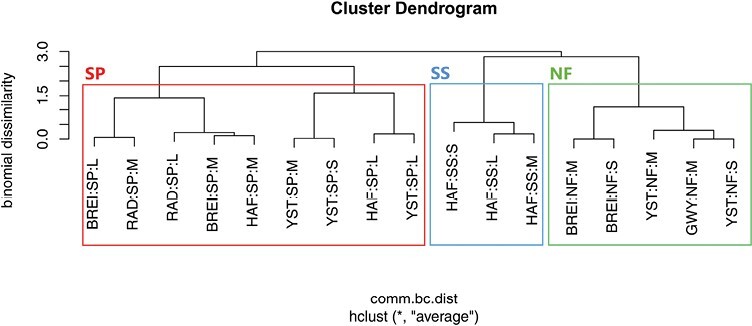
Cluster dendrogram generated with average-linkage algorithm and using species composition and showing similarities/dissimilarities between log samples. Tree species: SS = Sitka spruce, NF = Noble fir, SP = Scots pine. Forest sites: GWY = St Gwynno, BREI = Breidden, HAF = Hafren, RAD = Radnor, YST = Ystwyth. Log sizes: L = Large, M = Medium, S = Small.

None of the continuous environmental factors (i.e., diameter and bark thickness and distance to the ground) were significantly correlated to the communities found in the samples (respectively, *F*(1,15) = 1.025, *P* = 0.387; *F*(1,15) = 0.390, *P* = 0.813; and *F*(1,15) = 0.163, *P* = 0.939). However, tree species was significantly correlated to the differences in communities between samples (*F*(2,14) = 13.068, *P* = 0.001). Numerical classification separated the standing trap tree beetle communities based on tree species but not forest sites (Fig. 7).

### Beetle Communities in Log Piles

**Fig. 8.**
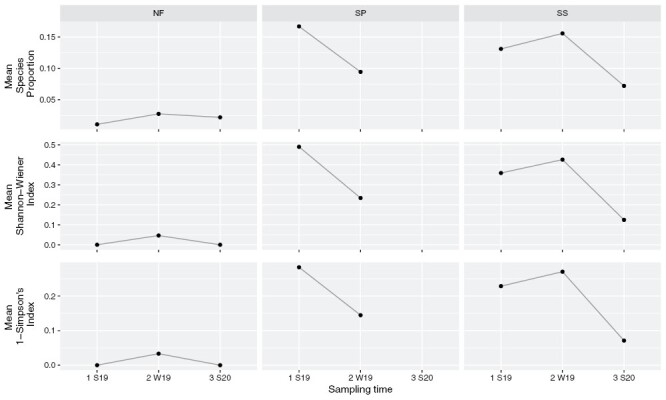
Mean Species proportion, Shannon–Wiener index, and 1-Simpson’s index plotted against tree species and for the different forest sites. Tree species: SS = Sitka spruce, NF = Noble fir, SP = Scots pine. Forest sites: GWY = St Gwynno, BREI = Breidden, HAF = Hafren, RAD = Radnor, YST = Ystwyth. Sampling time: S19 = Summer 2019, W20 = Winter 2020; S20 = Summer 2020.

Proportion of species, Shannon–Wiener Index, and Simpson’s index of diversity of beetle communities in the log piles from each of the 3 tree species followed the same pattern over the sampling period (Fig. 8). At the first sampling point (S19), tree species had a significant impact on the diversity measures. Noble fir had lower diversity than Scots pine (respectively for all measures: *P* = 0.001, *P* = 0.001, and *P* < 0.001) and Sitka spruce (*P* = 0.009, *P* = 0.008, and *P* < 0.001 – see Supplementary Table 2 for estimates). However, there was no significant difference between Sitka spruce and Scots pine diversity (*P* = 0.824, *P* = 1, and *P* = 1). At the second sampling point (W20), after 12 months, all 3 indices were significantly higher for Sitka Spruce than for Noble fir (*P* = 0.002, *P* = 0.002, and *P* < 0.001). Shannon–Wiener Index and Simpson’s index of diversity were not higher in Sitka spruce than in Scots pine (*P* = 0.212 and *P* = 0.184) but species proportion was (*P* = 0.017). Finally, Scots pine had higher species proportion than Noble fir (*P* = 0.025) but not Shannon–Wiener Index and Simpson’s index values (*P* = 0.208 and *P* = 0.242). At the third sampling point (S20), tree species did not significantly impact Shannon–Wiener Index and Simpson’s index though the difference between Noble fir and Sitka spruce was almost significant (*P* = 0.088 and *P* = 0.095). On the other hand, species proportion was significantly higher in Sitka spruce than in Noble fir (*P* = 0.026).

**Fig. 9.**
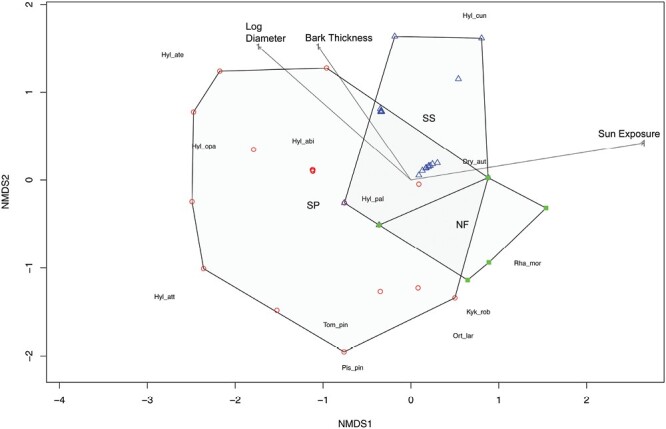
Nonmetric multidimensional scaling (NMDS) plot generated using species composition within log samples from log piles and showing similarities/dissimilarities between log samples (Triangles = Sitka spruce logs, Squares = Noble fir logs, Circles = Scots pine logs). Environmental variables were added and shown with arrows. The stress value of the analysis is 0.11.

The 2-D ordination plots (Fig. 9) indicated a much larger range of beetle community dissimilarities among samples from Scots pine (wider polygons) than among samples from Sitka spruce and Noble fir (more tightly clustered polygons). The beetle communities in Sitka spruce and Noble fir overlap each other partially, and both overlap a small part of the range of Scots pine, showing similarities in communities between tree species. Nevertheless, all 3 tree species comprised samples which were not overlapping with those of the other tree species, highlighting some specific communities for each tree species. The ordination plot also illustrates the association between beetles and environmental factors and Supplementary Table 3 shows the range of values for the continuous variables.

Tree species (*F*(2,63) = 12.994, *P* = 0.001), Ground level (*F*(1,64) = 11.755, *P* = 0.001) and sampling time (*F*(2,63) = 3.078, *P* = 0.013) all had a significant impact on the beetle community. Additionally, log diameter and bark thickness had a significant impact on the beetle community (respectively *F*(1,64) = 7.380, *P* = 0.001; *F*(1,64) = 7.472, *P* = 0.002).On the contrary, sun exposure and forest site did not have a significant impact on the beetle community present (respectively *F*(1,64) = 1.587, *P* = 0.206; *F*(4,61) = 0.246, *P* = 0.973).

Numerical classification did not separate the samples into clusters (Supplementary Fig. 1).

## Discussion

This study applied DNA barcoding of larvae to characterize insect communities present at different early decay stages (i.e., standing trap trees and log piles) in Scots pine, Sitka spruce, and Noble fir stands. The type of substrate was found to affect the beetle communities with those sampled from standing trap trees had lower diversity than those found in log piles. Tree species was found to be significant in structuring beetle communities at all stages of early succession, with Scots pine and Sitka spruce generally hosting more diversity than Noble fir. This sampling and DNA barcoding ID of larvae from standing trap trees or log piles provides precise knowledge on breeding substrate and nutrition requirements of individual species. While most of the beetle species found in the present study are not a source of concern for forestry, a few can be considered pests of conifers and were mainly found in trees in the initial stages of decay and early beetle community succession.

### Sampling of Larvae Provides Precise Knowledge on Breeding Substrate and Nutrition Requirements of Species

Sixteen different beetle species were found underneath the bark of the 3 tree species in the 2 experiments. This species diversity is comparable to results from other studies in Wales based on Sitka spruce stands only and using flight interception traps for sampling (Williams et al. 2017, 2021). Flight interception traps catch any adults flying in the forest and reveal the general spatial distribution of insect species, but direct sampling of larvae within trees provides precise knowledge on beetle-host tree associations, breeding substrate, and nutrition requirements of species. Studying larval stages is therefore improving the accuracy of ecological surveys (Köhler et al. 2022). This was confirmed by the lower number of broadleaf-associated species in our results in comparison to previous studies in Wales (Williams et al. 2017, 2021): only 2 species (*Kyklioacalles roboris* (Curtis) *and Rhagium mordax* (De Geer)) usually associated with broadleaf trees were recorded here in the sampled conifer species. Nevertheless, larvae are not usually sampled in monitoring projects due to the lack of taxonomic expertise and the difficulty of their identification (Köhler et al. 2022).

Because of their small size and cryptic lifestyle, collecting and studying bark and wood-boring larvae can be challenging. Uncovering the entire diversity of species feeding on the bark and the wood of dying and dead tree requires exhaustive sampling protocols. Because only the bark was sampled in our experiments, communities in branches, xylems, and stumps were not represented in our results. For instance, the number of Cerambycids found here was low (only 2 species, *R. mordax* and *M. minor*, respectively 17 and 13 individuals) and no ambrosia beetles or wood wasps were found (i.e., groups which bore into the xylem). Even though screening the xylem for beetles was not part of our sampling protocol, many species of wood-feeding larvae begin their lives in the phloem before moving into the sapwood (Stokland et al. 2012). Therefore, other factors such as low log moisture can also potentially account for the lack of highly abundant species previously found in Wales (e.g., *Trypodendron lineatum* (Olivier); Borkowski and Skrzecz 2016, Williams et al. 2017) but showing a preference for high moisture content. Although the diversity of species found could have been increased by increasing the diversity of substrate sampled, identification of immature stages feeding within trees, using DNA-based methods, is an effective way to precisely depict beetle and tree-host associations.

### Tree Species Strongly Shapes Beetle Community Composition at the First Stages of Early Succession

The species of conifer significantly impacted the beetle community composition and species diversity in both standing trap trees and log piles, though this effect seemed to be reduced at the end of the log pile experiment (S20). Our results support previous studies suggesting that host tree plays a fundamental role in structuring xylophagous communities (Bertheau et al. 2009) and that preferences of xylophagous species are more apparent and evident at earlier stages of wood decay (Stokland et al. 2012, Wende et al. 2017). Species colonizing freshly dead wood or dying trees need to overcome chemical barriers and tree species-specific compounds of hosts with more or less intact defence systems (Stokland et al. 2012, Wende et al. 2017). As the chemical and physical properties of the wood degrade, the logs of different tree species become available for a wider range of beetle species (Stokland et al. 2012). Although beetle species have host preferences many exhibit some level of plasticity (Bertheau et al. 2009). For instance, *R. mordax and K. roboris,* prefer broadleaf trees (Morris 2002, Heijerman 2004, Stüben 2015, Duff 2016), but were both found here in Noble fir logs. In addition to tree species, other factors such as log characteristics or surrounding environment appear to influence the species assemblages of recently dead and dying trees. For instance, bark thickness and log diameter had a significant impact on the beetle species composition found in the log piles.

### Lower Species Diversity Among Standing Trap Trees in Comparison With Log Piles

Fewer beetle species were caught and identified in the standing trap trees than in the log piles (respectively 6 and 12). This could be due to the lower number of logs sampled from standing trap trees versus piles (45 vs. 120, due to the delayed suitability for sampling of standing trap trees). However, logs from the standing trap trees experiment had a significantly lower proportion of species, lower Shannon–Wiener index, and lower 1-Simpson’s index than logs from the log pile experiment. Moreover, the accumulation curves suggest that this pattern would likely be confirmed by a more intensive sampling (i.e., plateauing of the curve representing the standing trap tree communities but not of the 1 representing the log pile communities). Despite the low species diversity, a high proportion of species found in standing trap trees (when compared to species in the log piles) have the potential to become significant pests or are closely related to beetle species with significant economic impact. For instance*, Cryphalus asperatus* is sometimes abundant in the UK while *C. piceae* is recorded as a nonestablished introduced species in the UK (Duff 2016). Neither is usually of economic importance, but they can both have a significant economic impact and kill healthy trees under certain circumstances (Justesen et al. 2020, Fiala and Holuša 2021). As another example*, Tomicus piniperda* is a significant pest of Scots pine (Lieutier 2002, Kerdelhue et al. 2006, Wainhouse and Inward 2016). *Pityogenes trepanatus* (Nördlinger) is of similar size to *P. chalcographus* (Linnaeus)*,* a major pest in Europe where it attacks Norway spruce and other European Pinaceae (Bertheau et al. 2012). *Molorchus minor and Pissodes pini are known to* damage trees of the genus *Pinus* and *Picea* that are severely stressed or dying (Day et al. 2007, Kenis and Hilszczanski 2007) and are regarded as a forestry pest of minor importance (Day et al. 2007, Duff 2016).

Ten species were identified only from log piles and were therefore associated with later stages of the early succession. Unlike the species caught in the standing trap trees, beetles associated with log piles can be beneficial as they reduce the amount of resources available for more damaging species. For instance, *H. palliatus* is a very competitive species, good at building up in numbers, which only uses dead material as it cannot deal with resin defenses. In contrast, *Hylastes* spp. (black pine beetles) and *H. abietis* were found in the log pile experiment and are pests of new coniferous plantings (Duff 2016). *H. abietis* is in fact the most important pest of reforestation in Northern and Western Europe, especially where a clear-cut plantation system is employed (Day et al. 2007). However, these species are not damaging trees when feeding in the bark but only when the adult weevils feed on the young stem of conifer transplants (Day et al. 2007). A particularly interesting finding of this study was the identification of *K. roboris* larvae developing underneath the bark of a Noble fir log. This revealed an unexpected habitat for this rare flightless weevil and highlighted that the ecology of this species is not well understood.

Generally, although lower species diversity was found in the standing trap trees, species identified from this experiment were more likely to cause damage to forestry trees in the future whereas species found in the log piles were more likely to be beneficial species, unless they could damage trees at other stages of their life cycle (e.g., *H. abietis*). Both experiments (standing trap trees and log piles) were useful to depict different aspects of the beetle community succession, and to identify pest species present on the different sites studied. The results reported here provide informative material for decisions on monitoring and management of potential pest species and on conservation and promotion of beneficial species. By better understanding the factors influencing the abundance and diversity of species, management techniques can be developed and tailored to limit the abundance of pest species while simultaneously increasing the abundance of secondary beetle species. The latter are very important for the dead wood decomposition process and prevent potentially damaging beetle species from building up in large numbers by competing with them for breeding substrate.

## Supplementary Material

nvad080_suppl_Supplementary_Figure_S1

nvad080_suppl_Supplementary_Material

## Data Availability

The data underlying this article can be accessed with accession numbers: OR255932-OR255946, OR255952-OR255956, OR255962-OR255964,OR255979-OR255997, OR256002-OR256006,OR256019, OR256031, OR256032, OR256034-OR256039.
