# Supplementary figures and images for: Time series DNA barcoding provides insight into factors influencing wood-boring and bark-feeding insect communities in Scots pine, Sitka spruce, and Noble fir stands
Source: Environ Entomol. 2023 Aug 17;52(5):802–13. doi: 10.1093/ee/nvad080 (PMC10578509; doi:10.1093/ee/nvad080)

binomial dissimilarity

0.0 0.5 1.0 1.5 2.0 2.5

# Cluster Dendrogram

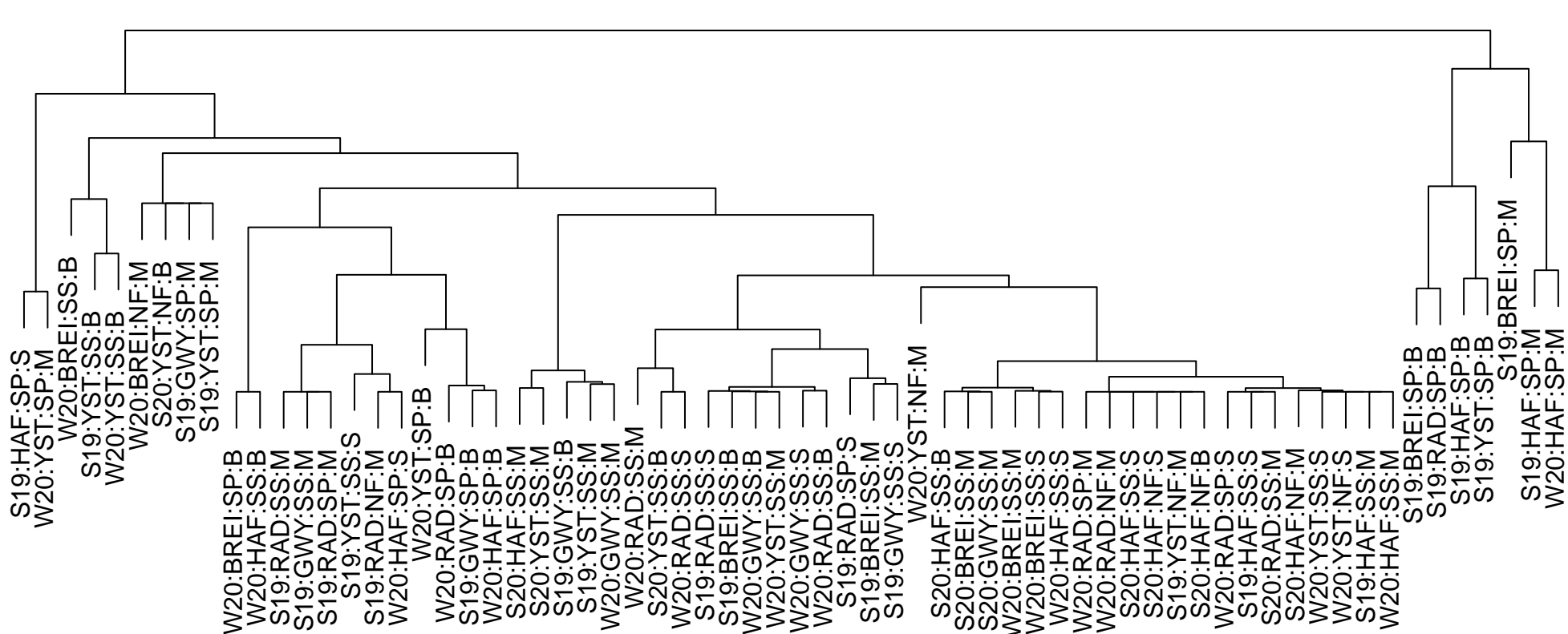

comm.bc.dist  
hclust (\*, "average")

Supplement: nvad080_suppl_Supplementary_Figure_S1 [file nvad080_suppl_supplementary_figure_s1.pdf]
