# Supplementary material for: Time series DNA barcoding provides insight into factors influencing wood-boring and bark-feeding insect communities in Scots pine, Sitka spruce, and Noble fir stands
Source: Environ Entomol. 2023 Aug 17;52(5):802–13. doi: 10.1093/ee/nvad080 (PMC10578509; doi:10.1093/ee/nvad080)
Supplement: nvad080_suppl_Supplementary_Material [file nvad080_suppl_supplementary_material.docx]

Supplementary Table 1: Minimum, maximum and mean values for the three continuously distributed environmental factors used in Log pile NMDS analysis (Standing trap trees).

| **Factor** | **Min** | **Mean** | **Max** |
| --- | --- | --- | --- |
| **Distance to the ground (m)** | 0.600 | 9.280 | 23.400 |
| **Log Diameter (cm)** | 5.700 | 19.790 | 35.800 |
| **Bark thickness (cm)** | 0.250 | 1.090 | 2.950 |

Supplementary Table 2: Estimate, Standard Error, t and p value for the tree species comparisons in the log pile experiment (at three sampling time). Bold values denote statistical significance at the p < 0.05 level.

| **Sampling Time** | **Tree Species comparison** | **Shannon–Wiener Index (Estimate, Standard Error, t and p value)** | **Simpson’s index of diversity (Estimate, Standard Error, t and p value)** | **Proportion of Species (Estimate, Standard Error, t and p value)** |
| --- | --- | --- | --- | --- |
| **S19** | SP > NF | β=0.345, SE=0.095 t(44)=3.635, **p=0.001** | β=0.226, SE=0.065 t(44)=3.483, **p=0.001** | β=-0.032, SE=0.006 t(44)=5.306, **p<.001** |
|  | SS > NF | β=0.265, SE=0.097 t(44)=2.743, **p=0.009** | β=0.185, SE=0.066 t(44)=2.806, **p=0.008** | β=0.029, SE=0.006 t(44)=4.752, **p<.001** |
|  | SP > SS | β=0.080, SE=0.097  t(44)=0.829, p=0.824 | β=0.041, SE=0.066  t(44)= 0.616, p=1 | β=0.003, SE=0.006 t(44)=0.462, p=1 |
| **W20** | SP > NF | β=0.141, SE=0.085  t(45)=1.668, p=0.208 | β=0.093, SE=0.058 t(45)=3.483, p=0.242 | β=0.015, SE=0.006 t(45)=2.626, **p=0.025** |
|  | SS > NF | β=0.281, SE=0.085, t(45)=3.323, **p=0.002** | β=0.194, SE=0.058 t(45)=3.315, **p=0.002** | β=0.030, SE=0.006 t(45)=5.395, **p<.001** |
|  | SS > SP | β=0.140, SE=0.085 t(45)=1.655, p=0.212 | β=0.101, SE=0.058 t(45)=1.728, p=0.184 | β=0.015, SE=0.006, t(45)=2.769, **p=0.017** |
| **S20** | SS > NF | β=0.094, SE=0.053 t(30)=1.778, p=0.088 | β=0.060, SE=0.034, t(30)=1.736, p=0.095 | β=0.011, SE=0.005, t(30)=2.368, **p=0.026** |

Supplementary Table 3: Minimum, maximum and mean values for the three continuously distributed environmental factors used in Log pile NMDS analysis (Log piles).

| **Factor** | **Min** | **Mean** | **Max** |
| --- | --- | --- | --- |
| **Bark thickness (cm)** | 0.100 | 0.970 | 2.850 |
| **Log diameter (cm)** | 4.500 | 19.170 | 35.600 |
| **Sun exposure (%)** | 28.000 | 55.000 | 97.000 |

Supplementary Figure 1: Cluster dendrogram generated with average-linkage algorithm and using species composition and showing similarities/dissimilarities between log samples (dots). Tree species: SS=Sitka spruce, NF=Noble fir, SP=Scots pine. Forest sites: GWY= St Gwynno, BREI=Breidden, HAF=Hafren, RAD=Radnor, YST=Ystwyth. Sampling time: S19= Summer 2019, W20=Winter 2020; S20=Summer 2020.
